# Parkinson’s Disease – the Debate on the Clinical Phenomenology, Aetiology, Pathology and Pathogenesis

**DOI:** 10.3233/JPD-130175

**Published:** 2013

**Authors:** Peter Jenner, Huw R. Morris, Trevor W. Robbins, Michel Goedert, John Hardy, Yoav Ben-Shlomo, Paul Bolam, David Burn, John V. Hindle, David Brooks

**Affiliations:** a Neurodegenerative Diseases Research Group, Institute of Pharmaceutical Sciences, School of Biomedical Sciences, King’s College, London, UK; b MRC Centre for Neuropsychiatric Genetics and Genomics, Cardiff University School of Medicine, Cardiff, UK; c Behavioural and Clinical Neuroscience Institute and Department of Psychology, University of Cambridge, Cambridge, UK; d MRC Laboratory of Molecular Biology, Cambridge, UK; e Department of Molecular Neuroscience, UCL Institute of Neurology, London, UK; f Faculty of Medicine and Dentistry, School of Social and Community Medicine, University of Bristol, Bristol, UK; g MRC Anatomical Neuropharmacology Unit, Oxford, UK; h Institute for Ageing and Health, Newcastle University, Newcastle upon Tyne, UK; i School of Medical Sciences, University of Bangor, Bangor, UK; j Faculty of Medicine, Centre for Neuroscience, Division of Experimental Medicine, Hammersmith Hospital, Imperial College, London, UK

**Keywords:** Parkinson’s disease, phenomenology, aetiology, pathology, pathogenesis, synucleinopathies

## Abstract

The definition of Parkinson’s disease (PD) is changing with the expansion of clinical phenomenology and improved understanding of environmental and genetic influences that impact on the pathogenesis of the disease at the cellular and molecular level. This had led to debate and discussion with as yet, no general acceptance of the direction that change should take either at the level of diagnosis or of what should and should not be sheltered under an umbrella of PD. This article is one contribution to this on-going discussion. There are two different themes running through the article - widening the definition of PD/LBD/synucleinopathies and the heterogeneity that exists within PD itself from a clinical, pathological and genetic per-spective. The conclusion reached is that in the future, further diagnostic categories will need to be recognized. These are likely to include - Parkinson’s syndrome, Parkinson’s syndrome likely to be Lewy body PD, clinical PD (defined by QSBB criteria), Lewy body disease (PD, LBD, REM SBD) and synucleinopathies (including LBD, MSA).

## INTRODUCTION

The understanding of what is meant by Parkinson’s disease (PD) has altered markedly over the past decade. The original definition by James Parkinson, and as subsequently modified by Jean-Martin Charcot, was descriptive [1]. Their clinical definition of the motor symptoms has served us well and has remained virtually unchanged over 200 years. Similarly, the description of pathology in substantia nigra, the effects of dopamine depletion in the caudate-putamen and the dramatic effects of dopamine replacement therapy demonstrated in the 1960 s have established a classical view of PD in the minds of generations of neurologists and geriatricians [2]. The clinical description of PD has defined a disorder which is likely to respond to dopaminergic therapies, and this therapeutic response in turn confirms the working diagnosis.

But with a new era of scientific discovery and more powerful investigative techniques, perhaps must come new thinking. The acceptance of the wide-spread α-synuclein pathology in PD, both in many brain areas outside the basal ganglia and in the periphery, has been linked to the concept of a progressive and possibly spreading disease process with a long pre-symptomatic phase, and to the importance of non-motor symptoms [3]. These may precede or follow the occurrence of motor symptomatology and from a central nervous system perspective take PD in to the domain of a neuropsy-chiatric disorder with anxiety, depression and cognitive change as key components of the syndrome [4].

The discovery of SNCA mutations and the role of genetics in PD have taken views on pathogenesis and clinical variation in PD to new levels [5]. Our concept of PD may need to be broadened to include new clinical syndromes, and conversely the variation within patients defined as having PD may mandate the definition of new subtypes which have a basis in clinical, genetic, pathological or neuro-imaging features. It may also become useful to define “synucleinopathy” as a clinical disease construct which includes conditions not currently defined as PD (e.g. dementia with Lewy bodies (DLB) and multiple system atrophy (MSA)), but excludes some conditions which may meet PD criteria (parkin disease and progressive supranuclear palsy (PSP)-parkinsonism), but have a different pathological basis [6]. This may involve the use of biomarkers, and analogously the clinical definition of Alzheimer’s disease has recently been revised to include biomarkers, which directly relate to the underlying disease process [7].

The complex nature of the motor and non-motor symptoms and the numerous different clinical mani-festations of PD might warrant a re-definition of the disease but this may lead to diagnostic confusion. The tip of the iceberg scenario for motor symptoms, the pyramid analogy and the elephant in the room illusion may suit text book definitions of PD but may not be particularly useful in a clinical setting as almost inevitably, it would be motor signs which lead a patient to seek medical advice. The Queen Square Brain Bank Criteria (QSBB) have been very effective at defining patients likely to respond to L-DOPA [8, 9]. We now need to build on the back of this bedrock of diagnostic excellence and provide new definitions that define patients likely to respond to disease modifying therapy, related to the underlying pathogenesis such as synuclein deposition, mitochondrial dysfunction or disorders of autophagy. So, do we now need to redefine what we refer to as PD or are we content with the clinical definition of the disease and then to modify it accordingly as knowledge advances?

From this perspective and as part on the on-going debate, we discuss the clinical phenomena of PD and how this reflects a diversity of disease expression that encompasses under the same umbrella, conditions not classically defined with PD and that stretch the definitions used to include both motor and neuropsychiatric consequences of the pathological changes that occur. Turning the issue on its head, we then look from the aetiological and genetic perspective at how this translates into the clinical phenomenology and again, to the conclusion that the shared pathogenic characteristics of a range of disorders lead to them being sheltered under the same umbrella.

## THE CLINICAL PHENOMENOLOGY OF PD AND ITS DIAGNOSIS

The common definition of clinical Parkinson’s syndrome or parkinsonism has rested on the presence of bradykinesia, with a progressive decrement in speed and amplitude of movement with one out of three features of rigidity, rest tremor or gait disturbance. Supporting features are important in the clinical definition of PD, and in diagnostic criteria, requiring additional components, such as progression, response to L-DOPA, asymmetry, rest tremor and more recently, the occurrence of non-motor symptoms [8]. On-going studies in apparently asymptomatic populations will show the patterns of appearance of motor and non-motor symptom predictive of a later clinical diagnosis of PD [10]. Importantly, the differentiation should be made between non-motor symptoms in established disease as opposed to the pre-motor phase of the illness. Neuropsychiatric disturbance, anosmia and constipation are the main candidates for relevant pre-motor symptoms. However the frequency of these symptoms in the normal ageing population may make it difficult to identify people who are likely to have Lewy body or α-synuclein related pre-motor symptoms with acceptable specificity. The difference in the rates of progression and the pattern of motor and non-motor symptom appearance between those with early onset versus late onset PD and those with akinetic-rigid PD and tremor dominant disease can be striking. A recent clinico-pathological study shows that patients with a tremor dominant presentation (sometimes referred to as benign tremulous Parkinson’s disease) have a slower disease progression than typical PD cases, but eventually develop the typical motor and non-motor features of PD, with substantia nigra Lewy body pathology [11]. Patients with non-tremor dominant presentations are more likely to develop dementia and markers of autonomic involvement such as orthostatic hypotension may also predict the development of dementia [12, 13]. REM sleep behaviour disorder is both a marker of pre-motor disease and associated with the development of cognitive impairment. In addition to differences in the progression of PD related to clinical sub-types some patients have genetic variants, which alter the rate of progression and disease phenotype. Recent data shows the PD patients carrying a single heterozygous mutation in the GBA gene have a faster clinical progression and develop cognitive impairment and dementia more quickly [14]. Clear distinctions can be made between very early onset PD (<18 years) and young onset cases (18–40 years) and those that form the bulk of the population with later onset PD (40–75 years) [15-17]. For example, dystonia is more common in earlier onset patients while tremor is more common in the elderly [18]. The ageing process or biological age are clear determinants of the onset of PD but also influence the appearance of non-motor symptoms, such as dementia. Indeed, there needs to be further investigation into the relative roles of disease progression as opposed to components of the overall syndrome that appear as part of the ageing process.

**Fig. 1:**
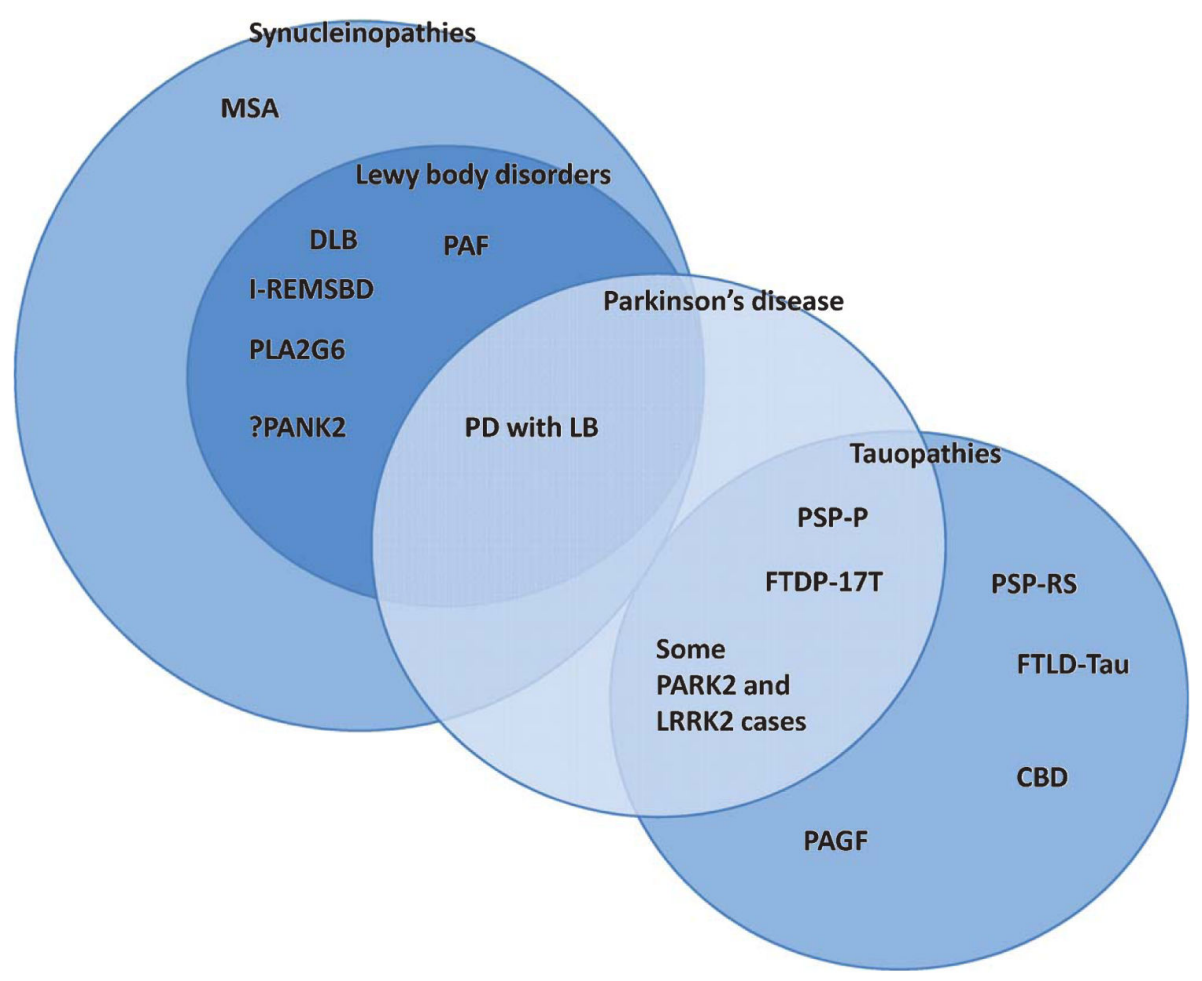
A concept of the overlapping pathologies and phenomenology of Parkinson’s disease.

It may be useful to develop criteria which encompass Lewy body disorders and more widely, synucleinopathies [19]. In clinical practice features such as rapid eye movement sleep behavioural disorder (REM SBD) and visual hallucinations are often used to support the clinical suspicion of PD, although these features may relate to early or late Braak stage disease (see Fig. 1). A broader concept of Lewy body disorders would include DLB, Lewy body PD and REM SBD related to Lewy body pathology (Braak stage 1 and 2) [20]. Although these are clinically separate entities, the common occurrence of Lewy pathology suggests that they could respond to similar therapies. MSA might be included in an overarching group of synucleinopathies, since its filamentous inclusions [21] are also made of α-synuclein [22-25] (Table 1). Filament morphologies differ between MSA and Lewy body diseases, suggesting that distinct conformers of assembled α-synuclein can give rise to different neurodegenerative diseases [23, 26]. Some cases with disease-causing mutations in *SNCA* exhibit neuropathological characteristics of both PD and MSA [27-30]. Sequence variation in *SNCA* is a risk factor for MSA, which is largely a sporadic disease [31, 32]. It follows that therapies targeting the abnormal aggregation of α-synuclein are also likely to be effective in MSA.

In some cases, additional biomarkers might be needed to improve the specificity of these diagnostic groups. For example, a young onset individual with dystonic features might be assessed for fronto-cortical symptomatology, cerebrospinal fluid biomarkers of α-synuclein and dopamine metabolism, and nigro-striatal imaging [33]. In contrast, older patients with akineticrigid disease should be monitored for cognitive decline and dementia. The distinction between the paths taken by early onset versus late onset cases needs further investigation and consideration when monitoring progression. DNA examination is likely to become a routine component of diagnosis and subtyping on a genetic basis may in the future be used to predict disease outcome. These additional measures would allow the definition of cohorts of patients that could form the basis for clinical trials of potential therapies and they would form more homogeneous groupings in which imaging could be used to map progression. Epidemiological investigations can be used to enable better risk stratification both at the pre-morbid stage or at early onset and hence enable targeting of any future disease modification approaches [34]. Issues such as ethnicity need addressing to determine their role in the expression of clinical parkinsonism as seen in the prevalence of MSA and cerebellar ataxias.

Accurately defining both disease commonality (“lumping”) and disease heterogeneity (“splitting”) is likely to need a multi-faceted approach using clinical, neuro-psychological, biochemical, imaging and pathological measures.

## PARKINSON’S DISEASE AS A NEUROPSYCHIATRIC DISORDER

Recognition of both the early and late occurrence of non-motor symptoms of PD is reflected clearly in the evolution of cognitive and neuropsychiatric components of the syndrome [35, 36]. Some of these deficits are at least as debilitating as the motor symptoms themselves, but they are less effectively managed at present and this currently represents an important unmet need. This field provides an excellent illustration of the change required in the diagnosis and approach to treatment in PD. In many respects, PD can be viewed as a neuropsychiatric disorder rather than a neurological disease.

It is commonly perceived that neuropsychiatric symptoms, progressive cognitive decline and dementia occur late in the course of PD and in the older patient population. Indeed, anxiety, depression, dopamine dysregulation syndromes leading to impulsive/compulsive behaviour (e.g. gambling addiction) and personality changes form common components of later stages of the illness but their recognition needs to be improved and treatments are currently largely ineffective [37-39]. However, it is clear that a history of anxiety and/or depression and an apathetic personality can also *precede* the onset of motor symptoms by many years. Depression and anxiety are major non-motor features of Parkinson’s disease; precise prevalence rates vary depending on precise diagnostic criteria and staging of Parkinson’s disease [40, 41]. Such symptoms are often associated with degeneration of the noradrenergic (locus coeruleus) and serotoninergic (midbrain raphé nuclei) systems but definitive evidence is lacking (see [42, 43]). The possible role of dopaminergic neuron loss in these symptoms also needs to be clarified [42]. It has been estimated that 26% of Parkinson’s disease patients are on pharmacotherapy for depression, [44] which has been claimed to be treated effectively using either tricyclic antidepressants or SSRIs or SNRIs [45] (but see also [46] for a negative metα-analysis). Suggestions that SSRIs can worsen PD motor symptoms have been shown to have only limited applicability [45, 47]. Possible advantages suggested for tricyclic antidepressants in terms of delaying need for dopaminergic medication in early Parkinson’s disease [48] or relative rapidity of ther -apeutic action [49] are offset by adverse side-effects of these drugs. Further research into the nature and treatment of affective disorder in Parkinson’s disease is warranted.

Cognitive deficits (especially associated with fronto-striatal dysfunction, see below) may also be present in early-in-the-course and in never-medicated patients [50]. These considerations make it clear that it is important to distinguish early and late cognitive and psychiatric sequelae of Parkinson’s disease. It is also relevant that the heterogeneity of cognitive deficits in the disease can be linked to different motor signs, early in the course of the disease. Thus, for example, cognitive deficits (whether of the fronto-striatal variety or frank dementia), are associated with late onset disease without tremor, whereas patients with tremor often escape such cognitive decline for many years [51]. These clinical presentations presumably arise from subtle differences in the regions and spread of the pathology and undoubtedly need further characterisation because of their possible status as ‘markers’ of later dementia. Thus, neuropsychiatric, and possibly cognitive, signs need to become part of the criteria in prodromal stages of PD that may be predictive of the nature of the future course of the disease.

The progressive nature of the cognitive and neuropsychiatric components is a reflection of the spreading pathology that is increasingly accepted to underlie PD. The loss of ascending chemical neurotransmitter systems from the isodendritic reticular core of the brain is probably a major component of pathology leading to depression, anxiety and cognitive decline. Early onset of these signs may indicate loss of noradrenergic input from the locus coeruleus and serotonin input from the raphé nuclei that can even precede changes in dopaminergic function.

Cognitive impairment is also a common (though under-recognised) component of early PD with alterations in executive function (e.g. inhibitory control, attention, working memory and planning) (e.g. [52, 53]). This ‘fronto-striatal’ syndrome is dopamine-dependent [54, 55] and may also be modulated by genetic polymorphisms affecting the dopamine systems, such as catechol-O-methyl-transferase (COMT) [56]. Frontal cortex levels of dopamine may initially increase in PD as a response to decreased dopamine levels in basal ganglia [57, 58]. However, this may be detrimental to cognition because of the well-known inverted U shaped curve linking impaired cognition to either under-activation or over-activation of dopamine systems with an intermediate level being required for optimal functioning (‘Yerkes-Dodson’ law [59, 60]). As a consequence, fluctuations in dopaminergic function increasingly affect cognition as PD pro-gresses. Dopaminergic medication can either reverse these impaired cognitive functions or actually produce deficits (perhaps due to ‘overdosing’ [61]), depending on the stage of illness, dose of medication and genetic status. On the other hand, there are certain cognitive deficits (e.g. in recognition memory, and some aspects of cognitive flexibility such as extrα-dimensional set-shifting) which are not necessarily modulated by dopamine, and may be mediated instead by deficits in other chemical transmitter systems (e.g. the coeruleal-cortical noradrenergic system in the case of set-shifting) (see [33]).

Fronto-striatal cognitive changes can occur independently of parkinsonian dementia and two separate syndromes may exist, dementia probably implicating cortical (i.e. extrα-striatal), dopamine-independent pathways (review; [33]). Parkinsonian dementia may develop later in the course or with age, and may arise at least in part from Lewy body or amyloid deposition in posterior cortical regions such as the parietal and temporal lobes. Characteristic signs of parkinsonian dementia (in contrast to the fronto-striatal dopamine-sensitive syndrome) are visuospatial constructional deficits (e.g. drawing, copying polygons) and recognition, semantic and episodic memory loss – these are commonly not ameliorated by dopaminergic medication [13] but do respond positively to cholinesterase inhibitors such as rivastigmine [62] consistent with the large reductions in cortical cholinergic markers in parkinsonian dementia which can be even greater than in Alzheimer’s disease [63]. This dementia may also co-occur with psychosis including visual hallucinations, which are worsened by dopaminergic treatment but may also be ameliorated by cholinesterase inhibitors (see [64]).

One complication is that, especially with increasing age, patients with PD may develop Aβ deposits and even the plaque and tangle pathology of Alzheimer’s disease [65]. Experimental studies have shown that Aβ can promote the accumulation of α-synuclein [66] and that α-synuclein aggregates can in turn induce the formation of tau pathology [67, 68]. Cortical Lewy pathology is the closest pathological correlate of dementia in PD [69].

## AETIOLOGY, PATHOLOGY AND PATHOGENESIS – WIDE AND DIVERSE

The aetiology of PD is often explained by environmental and genetic factors, with a postulated interaction between them. While perhaps partially correct, this is an ill-defined way of determining the cause of PD. Environmental or epidemiological studies have identified significant risk factors for PD, such as the exposure to pesticides, or protective entities, such as cigarette smoking and caffeine intake [70]. They have not helped with the identification of the primary mechanisms that underlie neurodegeneration in PD. However, we should not forget that it was clinical and epidemiological observations that led to the discovery of the nigral toxicity of MPTP [71] and the neurodegeneration caused by paraquat [72].

Age is the major known risk factor for the development of PD and it is crucial to understand its role. It is equally important to differentiate between early-onset and late-onset forms of disease. Risk stratification based on epidemiological investigations in the premorbid stage might be used to assess causative mechanisms and to relate these to future disease-modifying therapies. Much of the recent progress has come from an understanding of how some rare genetic causes of PD relate to the common Lewy pathology [73, 74]. This work has shown that mutations in *SNCA*, the α-synuclein gene, can cause PD [5, 30, 75-77, 78] and that α-synuclein is the major component of the Lewy pathology in all cases of PD [79, 80]. Clear links exist between these causes of disease and genetic risk factors identified in genome-wide association studies (GWAS) of idiopathic PD [81-83].

While the diagnosis of PD should remain based on clinical symptoms, the pattern and range of pathological changes are often used to differentiate PD from other forms of parkinsonism, including MSA and PSP (although, arguably, regional variation in atrophy on MRI provides an *in vivo* marker of pathology and can be used to assist with diagnosis). The new work makes it possible to subdivide PD further on mechanistic grounds.

Increasingly, Lewy pathology extending from the brainstem to the forebrain is accepted as occurring during PD [20]. It results in non-motor symptoms and involves many non-dopaminergic cell types, meaning that PD can no longer be viewed as a disease that predominantly affects the nigrostriatal dopaminergic system. Instead, PD is a multisystem disorder that affects many different regions of the nervous system [74].

The formation of α-synuclein inclusions is central to the development of PD [73, 74]. From this perspective, MSA and other synucleinopathies can also be included under the same pathological umbrella. Similar arguments revolve around the issue of the presence of tau inclusions. A valid view is to classify post-encephalitic parkinsonism, a pure tauopathy, separately, because tau inclusions are present in the substantia nigra in the absence of α-synuclein inclusions [84]. The same is true of cases with *MAPT* mutations and frontotemporal dementia and parkinsonism linked to chromosome 17 (FTDP-17T) [85-88], some of which can cause an asymmetric, akinetic-rigid syndrome whose initial stages are indistinguishable from PD. In other cases of PD and in some animal models thereof, both α-synuclein and tau inclusions are present [68, 89], suggesting an interaction between α-synuclein and tau [67].

Heterozygous mutations in the leucine-rich repeat kinase 2 (*LRRK2*) gene are commonly associated with parkinsonian motor symptoms and nerve cell loss in the substantia nigra [90, 91], but patients with these mutations exhibit either α-synuclein inclusions, tau inclusions or no apparent inclusions [92, 93]. Cases with α-synuclein inclusions manifest clinically as typical PD, whereas those with tau inclusions tend to fit the definition of the parkinsonian form of progressive supranuclear palsy (PSP-P). Disease penetrance in individuals with *LRRK2* mutations is age-dependent and ranges from 30–74%. These cases emphasise the relevance of genetic testing for the nosology of PD.

The concept of PD as a disease that originates in the enteric nervous system or in the brainstem, and involves the substantia nigra only later, widens its definition, which now includes the enteric, autonomic and central nervous systems [20, 74]. Acceptance of the concept that the pathological processes leading to PD permeate through the nervous system involves the assumption that the staging of brain material using α-synuclein immunoreactivity reflects a dynamic process that spreads reproducibly from an initial point of protein aggregation [94]. It is supported by the growing evidence that assembled α-synuclein behaves in a prion-like manner [95-98]. To test this concept further, it will be necessary to follow individuals who go on to develop motor symptoms longitudinally. Future characterisation of PD must include the imaging of aggregated α-synuclein. An alternative, but equally untested view, is that the pathological manifestations of PD reflect the temporal expression of a multifocal neurodegenerative process.

Individuals with mutations in the gene encoding glucocerebrosidase (*GBA*) are at increased risk of developing PD [99-101]. Defects in GBA have also been reported in idiopathic PD [102]. When compared to patients lacking mutations in *GBA*, patients with PD and *GBA* mutations have an earlier age of onset of disease and more severe non-motor symptoms, including autonomic dysfunction, neuropsychiatric symptoms and dementia [14, 103]. At autopsy, all cases with *GBA* mutations and PD have abundant Lewy pathology [104].

Defects in the repair of mitochondria give rise to recessive forms of juvenile-onset parkinsonism in individuals with loss-of-function mutations in the *Parkin* [105], *DJ-1* [106] and *PINK1* [107] genes. The abnormal aggregation of α-synuclein is not believed to be a significant feature of juvenile-onset parkinsonism [89]. However, additional cases of disease must be examined, before this conclusion can be universally accepted. It will be interesting to find out if the activity of the PINK1/Parkin pathway is impaired in idiopathic PD [108]. In the UK, mutations in *Parkin* are most commonly identified in early onset PD, followed by mutations in *PINK1* and *DJ-1* [109]. Individuals with pathogenic mutations in these genes have major abnormalities in mitochondrial function [110], suggesting that primary mitochondrial diseases can form part of the clinical PD umbrella. It raises the question of whether genetic testing should form part of the modified UK Brain Bank criteria, as suggested above. In idiopathic PD, 30% of cases showed a mitochondrial complex I defect at autopsy [111]. The differentiation between forms of PD with Lewy pathology and those without Lewy pathology provides an important distinction in the overall definition of what constitutes PD.

Two features that have emerged from GWAS are the importance of sequence variation in *HLA* [112, 113] and *MAPT* [81, 83, 114] as genetic risk factors for PD. The association with *HLA* suggests that activated microglial cells are involved in the pathogenesis of PD, in confirmation of previous findings [115]. It remains to be seen if this reflects a primary cause of PD or if it represents a secondary response to neurodegeneration. It implies that the role of microglial cells in “clean up” operations may be more significant than previously believed. Indeed, in MPTP-exposed individuals and MPTP-treated primates, microglial activation is present months or even years after toxin exposure [116, 117].

The identification of *MAPT* as a genetic risk factor for PD was surprising [114], given that PD is not a tauopathy. It is consistent with an interaction between tau and α-synuclein [67] and calls for further investigations to determine what role tau may play in the pathogenesis of Lewy pathology PD. *MAPT* has also been identified as a risk factor for MSA [118], suggesting that it may be a risk factor for all synucleinopathies. It has been suggested that the *MAPT* H1 haplotype is associated with the development of PD dementia [119]. Some patients with disease-causing *SNCA* mutations exhibit both α-synuclein and tau inclusions [88]. This genetic association also raises the issue of the relationship between PD and PSP-P, especially with regard to *LRRK2* mutations.

## CLOSING COMMENTS

There are two different themes running through the article - widening the definition of PD/LBD/synucleinopathies and the heterogeneity within PD itself from a clinical, pathological and genetic perspective. The current exclusion and supportive criteria are effective in defining PD related to LB disorders, but in many cases longitudinal follow-up and observation of the response to treatment may be needed to make a definitive diagnosis. It may take years to obtain clarity if there is, for example no rest tremor and the patient’s symptoms do not warrant L-DOPA treatment. The experience with potential disease modifying treatments in Alzheimer’s disease suggests that early diagnosis will become crucial. The addition of genetic testing, psychological profiling, biomarkers and the inclusion of other clinical features (for example anosmia, early cognitive change) may enable an earlier diagnosis of PD.

The current state of the art necessitates asking - what is PD as viewed in the 21st Century, when does it begin, how should it be defined? The QSBB define Parkinson’s syndrome with supportive inclusion and exclusion criteria helping to define Lewy body PD. Although some other pathologies have been described as leading to PD (such as some SCA gene mutations, *parkin-*positive parkinsonism and PSP– parkinsonism), it is clear that the QSBB criteria remain an extremely helpful and accurate clinico-pathological definition but that with time, further diagnostic groups will be needed.

These diagnostic categories are likely to include - Parkinson’s syndrome, Parkinson’s syndrome likely to be Lewy body PD, clinical PD (defined by QSBB criteria), Lewy body disease (PD, LBD, REM SBD) and synucleinopathies (including LBD, MSA). These categories do not correspond to classical clinico-pathological entities but are likely to be clinically and therapeutically useful. Our efforts should be directed towards consolidating these emerging diagnostic categories, and to developing new therapies for patients with these diseases.

## Figures and Tables

**Table 1:** Hierarchical classification of some neurodegenerative disorders based on protein deposition, cellular inclusions and clinico-pathological phenotype

Synucleinopathies	Lewy body disorders	Motor predominant Lewy body disorder
PD	PD	PD
DLB	DLB	
REMSBD	REMSBD	
Pure autonomic failure	Pure autonomic failure	
MSA		
